# Supplementary material for: Characterization of an undocumented CO2 hydrothermal vent system in the Mediterranean Sea: Implications for ocean acidification forecasting
Source: PLoS One. 2024 Feb 8;19(2):e0292593. doi: 10.1371/journal.pone.0292593 (PMC10852272; doi:10.1371/journal.pone.0292593)
Supplement: S2 Table — Species without values of frequency for all transects are those recorded only during the explorative survey. (DOCX) [file pone.0292593.s002.docx]

**Characterization of an undocumented CO2 hydrothermal vent system in the Mediterranean Sea: implications for ocean acidification forecasting**

D’Alessandro M, Gambi MC, Bazzarro M, Caruso CG, Di Bella M, Esposito V, Gattuso A, Giacobbe S, Kralj M, Italiano F, Lazzaro G. Sabatino G, Urbini L, De Vittor C.

|  | | | | |
| --- | --- | --- | --- | --- |
|  | **Percentage of frequency** | | | |
| **ANIMALIA** | **TR A** | **TR B** | **TR C** | **TR D** |
| [**Chordata**](http://www.marinespecies.org/aphia.php?p=taxdetails&id=1821) |  |  |  |  |
| *Aidablennius sphynx* (Valenciennes, 1836) |  |  |  |  |
| *Apogon imberbis* (Linnaeus, 1758) |  |  |  |  |
| *Chromis chromis* (Linnaeus, 1758) | 12 | 6.3 | 1.2 | 21.3 |
| *Coris julis* (Linnaeus, 1758) | 9.2 |  | 2.4 | 2.2 |
| *Diplodus annularis* (Linnaeus, 1758) |  |  |  |  |
| *Diplodus puntazzo* (Walbaum, 1792) |  |  |  |  |
| *Diplodus sargus* (Linnaeus, 1758) | 4.6 |  |  |  |
| *Diplodus vulgaris* (Geoffroy Saint-Hilaire, 1817) | 10 | 3.1 | 4.7 | 2.2 |
| *Epinephelus costae (*Steindachner, 1878) | 0.9 |  |  |  |
| *Epinephelus marginatus* (Lowe, 1834) | 0.9 |  |  |  |
| *Mullus barbatus* Linnaeus, 1758 | 5.5 |  | 1.2 |  |
| *Muraena helena* Linnaeus, 1758 |  |  |  |  |
| *Oblada melanura* (Linnaeus, 1758) | 2.8 | 2.1 | 3.5 | 5.6 |
| *Parablennius incognitus* (Bath, 1968) | 1.8 |  |  |  |
| *Sarpa salpa* (Linnaeus, 1758) |  | 4.2 | 5.9 |  |
| *Serranus scriba* (Linnaeus, 1758) | 2.8 |  |  |  |
| *Serranus cabrilla (Linnaeus, 1758)* |  | 2.1 |  |  |
| *Sparisoma cretense (*Linnaeus, 1758*)* |  |  |  |  |
| *Spicara maena* (Linnaeus, 1758*)* |  |  |  | 7.9 |
| *Symphodus roissali* (Risso, 1810*)* | 4.6 |  |  |  |
| *Symphodus tinca* (Linnaeus, 1758*)* | 17.4 |  |  |  |
| *Thalassoma pavo* (Linnaeus, 1758) | 6.4 | 2.1 | 1.2 | 1.1 |
| Trachinidae gen. sp. |  |  |  |  |
| *Trachinotus ovatus* (Linnaeus, 1758) |  |  |  |  |
| *Tripterygion* sp*.* |  | 2.1 |  |  |
| **Cnidaria** |  |  |  |  |
| *Balanophyllia (Balanophyllia) europaea* (Risso, 1826) |  |  |  |  |
| *Cladocora caespitosa* (Linnaeus, 1767) |  |  |  |  |
| *Pennaria disticha* Goldfuss, 1820 | 2.8 |  |  |  |
| **Crustacea** |  |  |  |  |
| Balanidae gen. sp. |  |  |  |  |
| *Percnon gibbesi* (Milne Edwards, 1853) |  |  |  |  |
| **Echinodermata** |  |  |  |  |
| *Holothuria* sp. | 0.9 |  |  |  |
| *Paracentrotus lividus* (Lamarck, 1816) |  |  |  |  |
| *Arbacia lixula* (Linnaeus, 1758) |  |  |  |  |
| **Mollusca** |  |  |  |  |
| *Cerithium vulgatum* Bruguière, 1792 | 0.9 |  |  |  |
| *Chamelea gallina* (Linnaeus, 1758) |  |  |  |  |
| *Columbella rustica* (Linnaeus, 1758) |  |  |  |  |
| *Conus ventricosus* Gmelin, 1791 |  |  |  |  |
| *Donax trunculus* Linnaeus, 1758 |  |  |  |  |
| *Hexaplex trunculus* (Linnaeus, 1758) |  | 4 |  |  |
| *Patella caerulea* Linnaeus, 1758 | 1.8 |  |  |  |
| *Patella rustica* Linnaeus, 1758 |  |  |  |  |
| *Patella ulyssiponensis* Gmelin, 1791 |  |  |  |  |
| *Phorcus articulatus* (Lamarck, 1822) |  |  |  |  |
| *Phorcus turbinatus* (Born, 1778) |  |  |  |  |
| *Pinna rudis* Linnaeus, 1758 |  |  |  |  |
| *Stramonita haemastoma* (Linnaeus, 1767) | 0.9 |  |  |  |
| *Tarantinaea lignaria* (Linnaeus, 1758) |  |  |  |  |
| *Vermetus triquetrus* Bivona-Bernardi, 1832 | 3 |  | 6 |  |
|  |  |  |  |  |
| **Polychaeta** |  |  |  |  |
| *Serpula vermicularis* Linnaeus, 1767 |  |  |  |  |
| **Porifera** |  |  |  |  |
| *Crambe crambe* (Schmidt, 1862) | 2.8 | 1.0 | 3.4 | 1.1 |
| *Sarcotragus* sp. Schmidt, 1862 | 20.2 | 73 | 77.7 | 7.9 |
| *Ircinia irregularis* (Poléjaeff, 1884) | 5.5 |  |  |  |
| **Ochrophyta** |  |  |  |  |
| *Padina pavonica* (Linnaeus) Thivy, 1960 | 14.7 | 78.1 | 89.4 | 33.7 |
| *Halopteris scoparia* (Linnaeus) Sauvageau, 1904 | 47.7 | 32.6 | 71 |  |
| **PLANTAE** |  |  |  |  |
| **Rhodophyta** |  |  |  |  |
| *Corallina elongata* (Ellis & Solander) Hind & Saunders, 2013 | 7.3 | 32.3 | 71.8 |  |
| *Jania* cf *rubens* Lamouroux, 1812 | 28.4 | 58.3 | 72.9 | 13.5 |
| **Chlorophyta** |  |  |  |  |
| *Halimeda tuna* (Ellis & Solander) Lamouroux, 1816 |  |  |  |  |
| *Codium bursa* (Olivi) C. Agardh, 1817 |  |  |  |  |
| *Anadyomene stellata* (Wulfen) C. Agardh, 1823 | 2.8 | 7.3 | 7.1 | 3.4 |
| *Caulerpa cylindracea* (Sonder, 1845) |  | 1.0 |  |  |
| *Caulerpa taxifolia var. distichophylla* (Sonder) Verlaque, Huisman & Procaccini, 2013 |  |  |  |  |
| [**Tracheophyta**](http://www.marinespecies.org/aphia.php?p=taxdetails&id=596326) |  |  |  |  |
| *Posidonia oceanica* (Linnaeus) Delile*,* 1813 | 1.8 |  |  |  |
