# Supplementary material for: Characterization of an undocumented CO2 hydrothermal vent system in the Mediterranean Sea: Implications for ocean acidification forecasting
Source: PLoS One. 2024 Feb 8;19(2):e0292593. doi: 10.1371/journal.pone.0292593 (PMC10852272; doi:10.1371/journal.pone.0292593)
Supplement: S1 Fig — Fig 1. Draft of the Gas Output Instrument to measure the air-water gas exchange. Fig 2. INGV researcher positioning the Gas output instrument on a sampling site off the San Giorgio vents. (DOCX) [file pone.0292593.s003.docx]

**Characterization of an undocumented CO_2_ hydrothermal vent system in the Mediterranean Sea: implications for ocean acidification forecasting**

D’Alessandro M, Gambi MC, Bazzarro M, Caruso CG, Di Bella M, Esposito V, Gattuso A, Giacobbe S, Kralj M, Italiano F, Lazzaro G. Sabatino G, Urbini L, De Vittor C.

**Gas Output Instrument**

The instrument is composed of three main parts (S3 Fig 1):


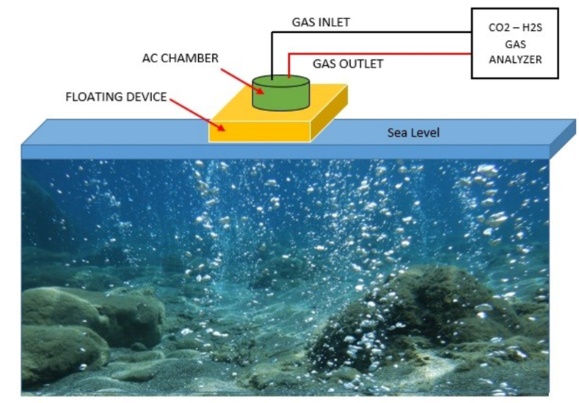


**S3 Fig 1. Draft of the Gas Output Instrument to measure the air-water gas exchange.**

1. Floating device: a platform allowing the chamber to float on seawater

2. AC chamber: accumulation chamber equipped with a pump that transfers gases to gas detector.

3. Gas analyser: CO_2_ (IRGA - CO2 Infrared Gas Sensor Gascard NG 10%) and electrochemical sensors (0 - 100 ppm) for H_2_S. During the measures, the chamber was sealed at the water surface, and continuous measurements of CO2 and H_2_S were made for approximately 3 min immediately after deployment (S3 Fig 2).


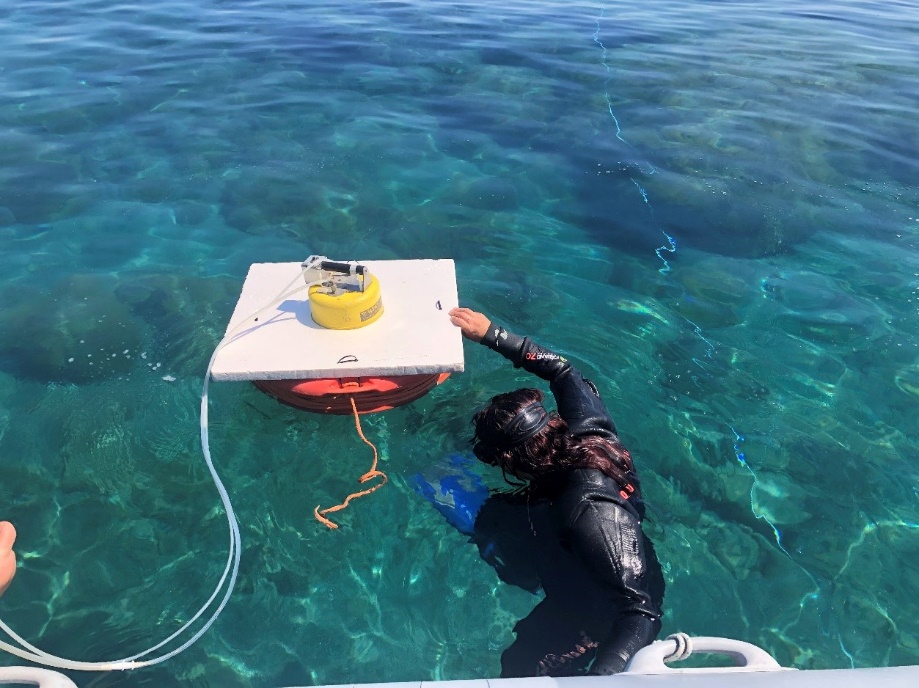


**S3 Fig 2. INGV researcher positioning the Gas output instrument on a sampling site off the San Giorgio vents.**

After a given time of pump activation, the value of gases concentration inside the probe reaches a steady value.
